# Synergistic effects of multiple “good agricultural practices” for promoting organic carbon in soils: A systematic review of long-term experiments

**DOI:** 10.1007/s13280-025-02188-8

**Published:** 2025-05-27

**Authors:** Raül López i Losada, Katarina Hedlund, Neal Robert Haddaway, Ullrika Sahlin, Louise E. Jackson, Thomas Kätterer, Emanuele Lugato, Helene B. Jørgensen, Per-Erik Isberg

**Affiliations:** 1 Centre for Environmental and Climate Science (CEC), Lund University, 223 62 Lund, Sweden; 2 Department of Biology, Lund University, 223 62 Lund, Sweden; 3 Harper Adams University, Newport, Shropshire, TF10 8NB UK; 4 Department of Land, Air and Water Resources, University of California Davis, One Shields Avenue, Davis, CA 95616 USA; 5 Department of Ecology, Swedish University of Agricultural Sciences, P.O. Box 7044, 750 07 Uppsala, Sweden; 6 European Commission, Joint Research Centre (JRC), Ispra, Italy; 7 Department of Statistics, Lund University, 220 07 Lund, Sweden

**Keywords:** Amendment, Carbon sequestration, Crop rotation, Fertiliser, Meta-analysis, Tillage

## Abstract

Loss of soil organic carbon (SOC) from farmland is a key threat to the capacity of soils to provide ecosystem services and exacerbates climate change. In alignment with a published protocol, we conducted a review and meta-analysis of time series of SOC measurements in long-term agricultural experiments to study absolute SOC changes under different agricultural management regimes. Our results show that SOC in the upper 30 cm layer generally declined across long-term experiments (214 time data series in arable land across 23 sites in temperate to cold regions), but reducing tillage, adding organic amendments, diversifying crop rotations and avoiding bare fallows reduced losses. Furthermore, a net increase in SOC content is achieved when combining all four interventions. Applying multiple strategies for SOC preservation can unleash the potential for agricultural land to become a carbon sink while safeguarding agricultural yields for future generations.

**Supplementary Information:**

The online version contains supplementary material available at 10.1007/s13280-025-02188-8.

## Introduction

Loss of soil organic carbon (SOC) from farmland is identified as a key threat to the capacity of soils to provide ecosystem services for agriculture and exacerbates climate change (Brady et al. 2019; Oldfield et al. 2019; Kätterer and Bolinder 2024). In the US, it is estimated that soils may have lost 30% to 50% of the SOC they contained before agriculture was established (Kucharik et al. 2001). Monitoring of SOC in Europe also suggests that stocks are being depleted, especially in agricultural land dominated by cereal crops (Mestdagh et al. 2009; De Rosa et al. 2024). Agricultural soil management promoting SOC accrual offers therefore the potential to enhance soil fertility while contributing to climate change mitigation efforts (Bolinder et al. 2010; Brady et al. 2015; Lal et al. 2021).

The evaluation of SOC accrual across regions is often confounded by the presence of a broad suite of management practices and distinct soil types when combining monitoring data from areas under different land uses, which must be considered with modelling or statistical analyses (Taghizadeh-Toosi et al. 2014). Meanwhile, local-scale agricultural experiments provide valuable resolution in efforts to detect changes in SOC stocks following the implementation of specific management practices compared to a control. Such experiments often only examine treatment effects at a single point-in-time, which precludes estimation of net SOC change rates, defined as the net emissions/removal of SOC for a given treatment over time (Sanderman and Baldock 2010; Muñoz et al. 2024). Yet, a single management intervention can show a relative increase in SOC in comparison with a conventional practice but still result in a net SOC loss over time (Sanderman and Baldock 2010).

In contrast, SOC stocks or concentrations are often recorded over many years in existing long-term agricultural field experiments (Haddaway et al. 2015). Indeed, evidence exists from long-term experiments in Europe and North America that net SOC change rates can still be negative even when straw is returned (Droste et al. 2020), low-intensity tillage is practiced (Bremer et al. 1995), or crop rotations are diversified (Nilsson et al. 2023). However, agricultural management in long-term field experiments often reflects normal practices in the regions where they are established and are generally not designed to compare multiple management systems (Haddaway et al. 2015). At the same time, managing arable land with practices for SOC preservation comprises a range of options that are not mutually exclusive, such as reduced tillage and use of organic amendments (Francaviglia et al. 2023). This stresses the relevance of identifying suites of practices that effectively enhance SOC to mitigate climate change and safeguard yields for future generations. In alignment with a published protocol (Haddaway et al. 2016), we conducted a review and meta-analysis of time series of SOC measurements in long-term agricultural experiments to evaluate whether SOC is being lost or sequestered under a broad range and combination of practices comprising tillage, use of fertiliser and organic amendments, and crop rotations.

## Materials and methods

Here we describe a systematic review to identify time series of SOC measurements in long-term experiments spanning at least 30 years under various agricultural management practices. In addition, our work includes a meta-analysis of net SOC change rates from the evidence base found in the literature review to determine the effect of different and combined agricultural management practices on SOC.

### Literature search

The search for experimental field studies potentially holding relevant time series was previously conducted as part of a set of published reviews. Firstly, a systematic map was carried out in September 2013 for interventions relating to amendments, fertiliser, tillage, and crop rotations (Haddaway et al. 2015). This search was then updated with findings from a systematic review of tillage (Haddaway et al. 2017), and a scientific report reviewing crop rotations (Brady et al. 2021) performed in alignment with a protocol established by Land et al. (2017). The combined outcome from the three reviews consisted of 615 unique publications containing 795 potentially relevant field studies, which are described in the aggregated meta-database of agricultural practices and SOC change data (Supplementary Information file S2). No specific update was performed in relation to organic amendments, but this intervention was categorised in the meta-database whenever present. A decision was made by the review team that the marginal added value would not warrant the effort to update the search for relevant publications after 2019. Detailed information regarding search strings, databases consulted and dates for each search is included as a supplementary information file (S6).

### Inclusion criteria for the time series data

The 795 studies present in the aggregated meta-database were reviewed for SOC time series with a data recording period equal to or over 30 years containing at least three temporal replicates. Our definition of long-term experiments (at least 30 years) was driven by the need for data with a high degree of power in relation to curve fitting, i.e., 20 years to allow substantial change in SOC due to management plus an additional 10 years that should pass before changes can be detected (Smith et al. 2014; Haddaway et al. 2017). In detail, the inclusion criteria for time series data in this systematic review were as follows:

- *Relevant subject:* Arable soils in agricultural regions with favourable climatic conditions to grow wheat, which are defined as those within the warm temperate climate zone (fully humid and summer dry; i.e., Köppen-Geiger climate classification: Cfa, Cfb, Cfc, Csa, Csb, Csc, including also bordering semi-arid regions BSk), and the snow climate zone (fully humid; i.e., Köppen-Geiger climate classification: Dfa, Dfb, Dfc). This general criterion ensures that the agricultural systems included in this review are reasonably similar.
- *Relevant interventions:* Any described agricultural management practice relating to different types, methods or amounts of fertiliser and organic amendments (including manure, crop residues, green manure, lime, sewage sludge, processed wood, peat/sediment, domestic waste/compost, bone meal/animal products); tillage intensity (no tillage/direct drill, reduced/conservation tillage, rotational/occasional tillage, conventional tillage, subsoiling); and crop rotations (monocultures, different crop sequences and rotation lengths, legumes, fallow, energy crops, annuals, perennials).
- *Relevant outcomes:* Soil C measures, including SOC, total organic carbon (TOC), total carbon (TC) where soils are shown to lack carbonates, and soil organic matter (SOM). All C measures may be expressed either as a concentration (e.g., g/kg or %) or as a stock (e.g., Mg/ha).
- *Relevant study types:* Studies must have examined interventions that have lasted at least 30 years to ensure that changes in SOC are detectable (Smith 2004) and to allow time series to be used in nonlinear estimates of net decay or accumulation rates. Studies must involve at least three outcome measurements across this period. Data had to be available for the specific interventions and not represent average data across different treatments. Studies concerning laboratory and mesocosm (i.e., greenhouse) experiments or modelling exercises were excluded unless they also presented primary data from field studies.

In addition, all records of relevant time series were screened for duplicates (both duplicate records in our database and dual publication). In some instances, more than one publication referred to the same experimental study but focused on complementary aspects, thereby possibly aggregating the same spatial replicates differently. In these cases, we included all contrasting time series from a single experimental study, as we considered them to provide complementary insight on the underlying primary data. Ultimately, independent data points were the experimental study (over time) rather than publications or bibliographic records.

### Data extraction strategy for time series

Within the 795 studies described in the aggregated meta-database, a total of 214 SOC time series from 41 publications spread across 20 locations fulfilled all criteria to be included in the meta-analysis. All time series present in each study were reviewed according to the inclusion criteria individually, leading to studies where only a fraction of their time series was included in our meta-analysis. The following information was extracted as meta-data for all included time series: citation; study location (country, site, climate zone, latitude, and longitude); soil type (texture classification or percent clay/silt/sand); study description (start year, duration, agricultural practices investigated, experimental design); and sampling strategy (spatial and temporal replication, soil sampling depth, C measurement method). Climate zone and soil type were regarded as key sources of heterogeneity and treated as potential modifiers to account for significant differences between studies as described in Sect. “Meta-analysis of net SOC change rates”. Interventions were assigned categorical values within five groups of management practices (Table 1). In addition, quantitative data (i.e., study findings) were described (outcome type, units, data location) and extracted in full.

**Table 1:** Categorical values assigned to the different groups of management practices. *A field is considered as a monoculture if it has the same crop every year, and as a rotation if it has any combination of different crops across the years. Rotations are further classified based on whether they contain grasses or legumes (G/L). **Fallow comprises experimental plots where soil is tilled and left bare, either continuously or as a summer fallow

Management practices	Categorical values
Tillage	High-reduced
Rotation*	Monoculture-rotation-rotation with grasses or legumes (G/L)
Fallow**	With–without
Amendments	None-organic
Inorganic fertiliser	Without–with

The studies in this systematic review were appraised in four domains: spatial (true) replication, temporal replication, study duration and soil sampling depth. Based on a classification of study validity included in Supplementary Information file S5 (Table S5.8), each domain was assigned an appraisal score of ‘?’ (missing information), 0 (low), 1 (medium), or 2 (high). Scores for the individual domains were summed, and those studies that achieved a summed score above 4 (maximum of 8) were given an appraisal category of ‘high’ validity, while those of 4 or below were assigned a ‘low’ validity. Studies where any category received a ‘?’ were classified as ‘Unclear’. The data extracted in full for the 214 SOC time series included is available as a supplementary information file (S1).

### Calculation of net SOC change rates for time series

A preliminary evaluation of curve fit was conducted to identify a general model to estimate net rates of SOC change over time across all time series, while finding the best fit for each time series was beyond the scope of this study. A log-linear model was selected based on this evaluation because it provided better fits compared to linear and logistic alternatives. We thereafter fitted log-linear curves to each available time series, which yielded a net rate of SOC change (i.e., the rate of change per year within each time series) and a standard error of the rate. The log-linear model function employed was:

$$\text{ln } y=a*t+b$$

*where y:* SOC level (as a stock or concentration), *t*: time (years from 0, i.e., the time of the first measurement), *b*: intercept parameter for initial SOC level at *t* = 0, *a:* parameter for the yearly net rate of change expressed in years^−1^.

The models were fitted using the lm function in R (R Development Core Team 2010). For each curve fit, intercept and rate of change were recorded along with their standard errors, in addition to measures of goodness of fit (R-square) and the* p* value for a hypothesis test if the rate of change was different from zero (see supplementary information file S3). One benefit of our meta-analysis approach is that the combination of studies reporting concentration and stock data did not pose any real challenges for incorporating them together in a synthesis, given that net SOC change rates were estimated individually for each time series.

### Meta-analysis of net SOC change rates

The impact of interventions on SOC was investigated across time series with different types of management by estimating the average effect of interventions with a meta-analysis with categorical factors. For this purpose, we used a mixed effects model on the estimates of net rates of SOC change from individual time series and with the associated standard errors of the mean. The individual studies were weighted according to the inverse-variance method using the *rma.mv* function within the *metaphor* package in R (Viechtbauer 2010). A unique study ID was given to all time series data within the same experimental facility and included as a random moderator to account for non-independent geographic (e.g., soil texture, climatic conditions), and experimental variation.

Effects were derived from the meta-analysis for each intervention individually, and for all possible combinations of pairs of practices comprising inorganic fertiliser application, tillage, rotation, bare fallow, and use of amendments. The level of aggregation used in the meta-analysis was decided considering the number of available studies and the distribution of interventions as main categorical factors.

### Sensitivity analysis of the quality of included studies

For sensitivity analysis, we used scoring from the critical appraisal to evaluate the impact of study quality on the results. To this end, we rerun the meta-analysis excluding data sets from research articles classified with an unclear or low score. The average effect of each intervention when estimated with a meta-analysis that only included time series with a high appraisal score aligned well with the main findings of this study, which indicated robust results regarding the validity aspects considered within the score. However, the exclusion considerably reduced the amount of available time series, which is reflected in larger ranges for the confidence intervals of the average effects of interventions. Results for this analysis are reported in the supplementary material (Fig. S5.6).

## Results

### Synthesis of the evidence base

**Fig. 1:**
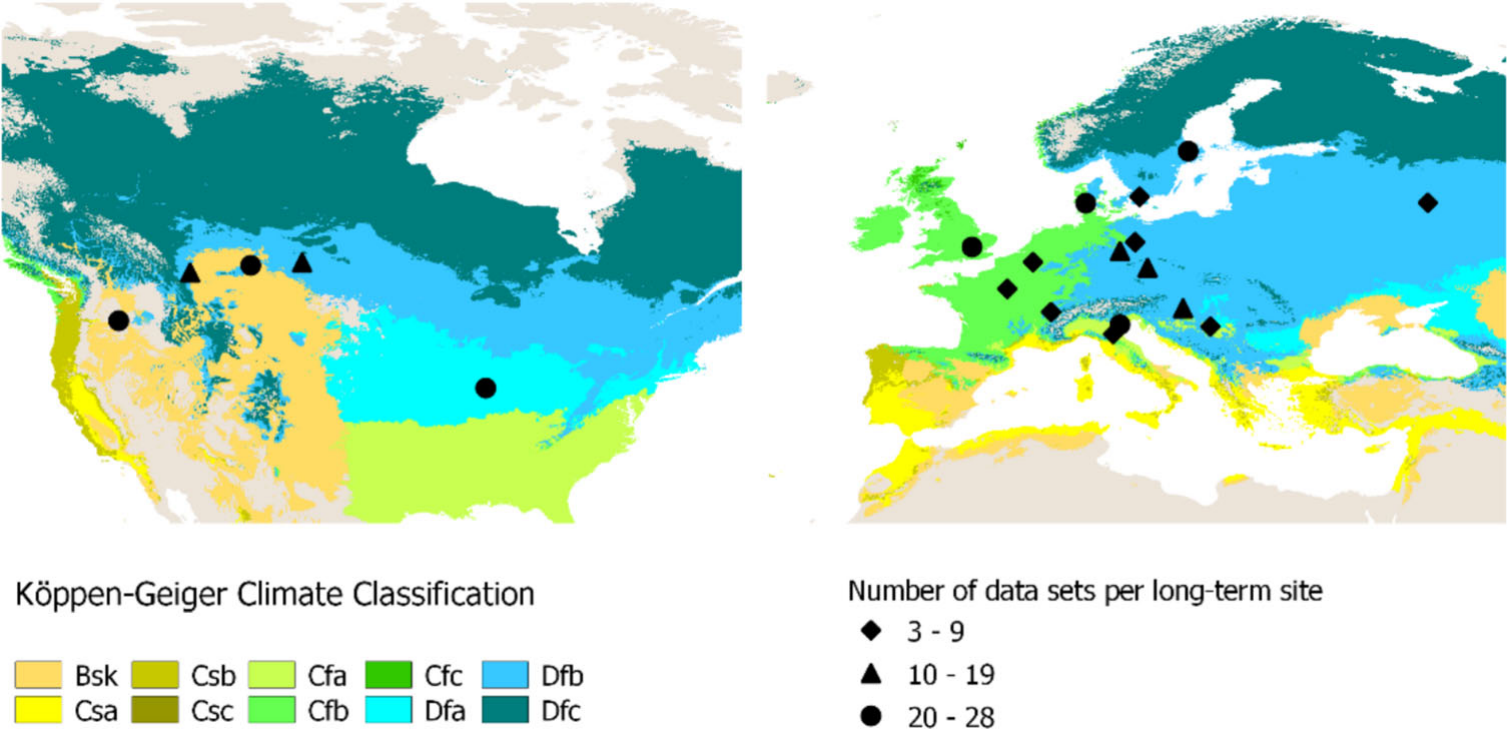
Location of long-term experiments included in this review. Climate classification from Beck et al. (2018)

Most time series included in this review were initiated between 1950 and 1970 (60%) and were 30–55 years in length (75%), while 31 time series were over 100 years old. The majority (66%) reported data for 3 up to 11 sampling dates, with a small number (5%) for more than 30. Core sampling varied in depth between 0–15 and 0–40 cm, with most samples (70%) ranging between 0–20 cm and 0–40 cm. Included experiments were spread across 13 countries in Europe and North America (Fig. 1). Italy and Sweden were the most frequently represented countries in number of time series, with the US, Denmark, and Canada following closely.

**Fig. 2:**
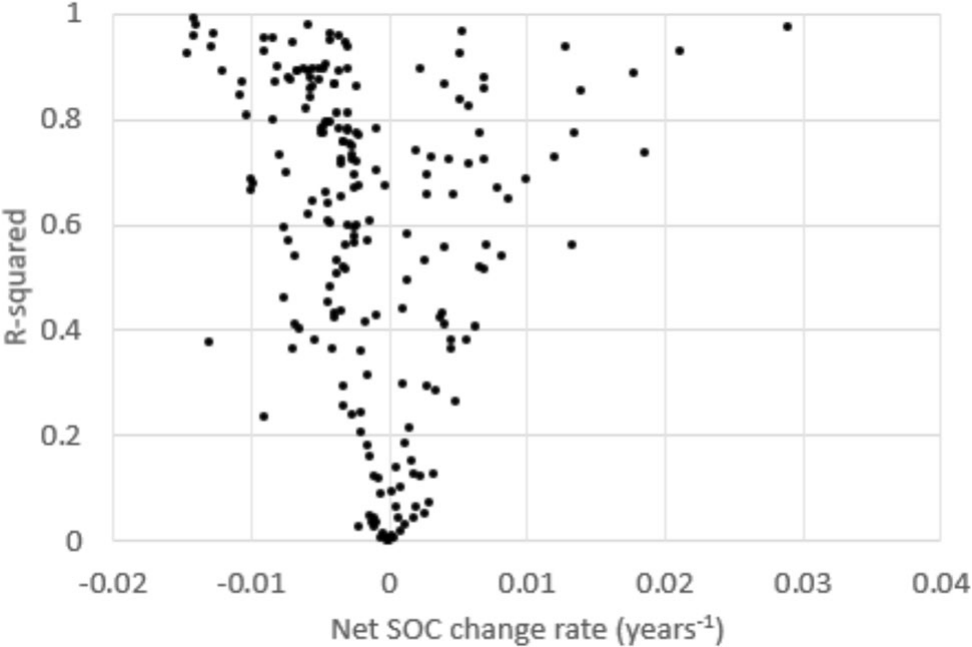
Fitted SOC change rate parameter and R-square statistics across included time series

SOC change rates across the 214 time series fitted to our log-linear model varied between − 0.015 and 0.029 (Fig. 2), with those experiencing a SOC loss being predominant (66%). A large majority of the time series (77%) showed an R-square of 0.3 or higher, while more than half (55%) showed an R-square of 0.6 or higher. Our log-linear model thus explained a considerable part of the variation in the response variable for most data sets. Furthermore, most time series with a low R-square showed SOC change rates around zero, indicating time-independent variability of SOC rather than poor model choice. The share of time series with a close fit was significantly higher for negative SOC change rates, which may indicate that our log-linear model is better suited to explain SOC declines than increases.

**Fig. 3:**
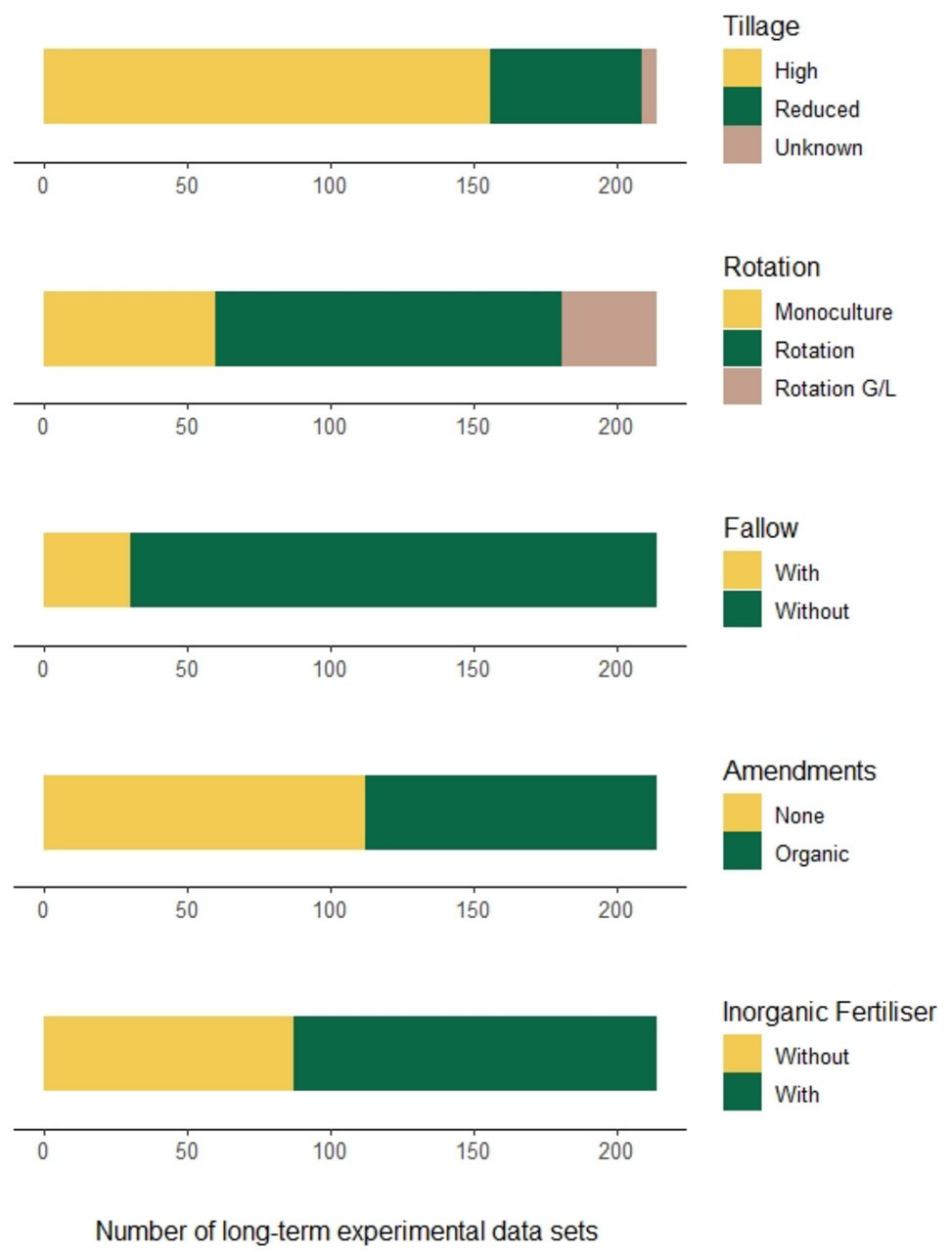
Long-term experimental data sets for 214 time series categorised based on their management practices regarding tillage, crop rotation, fallow, amendments, and inorganic fertiliser

The available evidence was distributed heterogeneously across considered interventions within the five management groups (i.e., tillage, crop rotation, use of amendments, inorganic fertiliser, and fallow), with some treatments contributing significantly more time series than others (Fig. 3). A matrix of Chi-squared tests performed on all pairs of possible interventions revealed that the association of different treatments was not always random, which is likely as experimental setups are designed to reflect common combinations of practices by farmers (Table S5.3). Five time series were disregarded from the meta-analysis given that it was not possible to determine their tillage intensity, thus effectively reducing our pool of evidence to 209 time series.

### Effects of individual management interventions

**Fig. 4:**
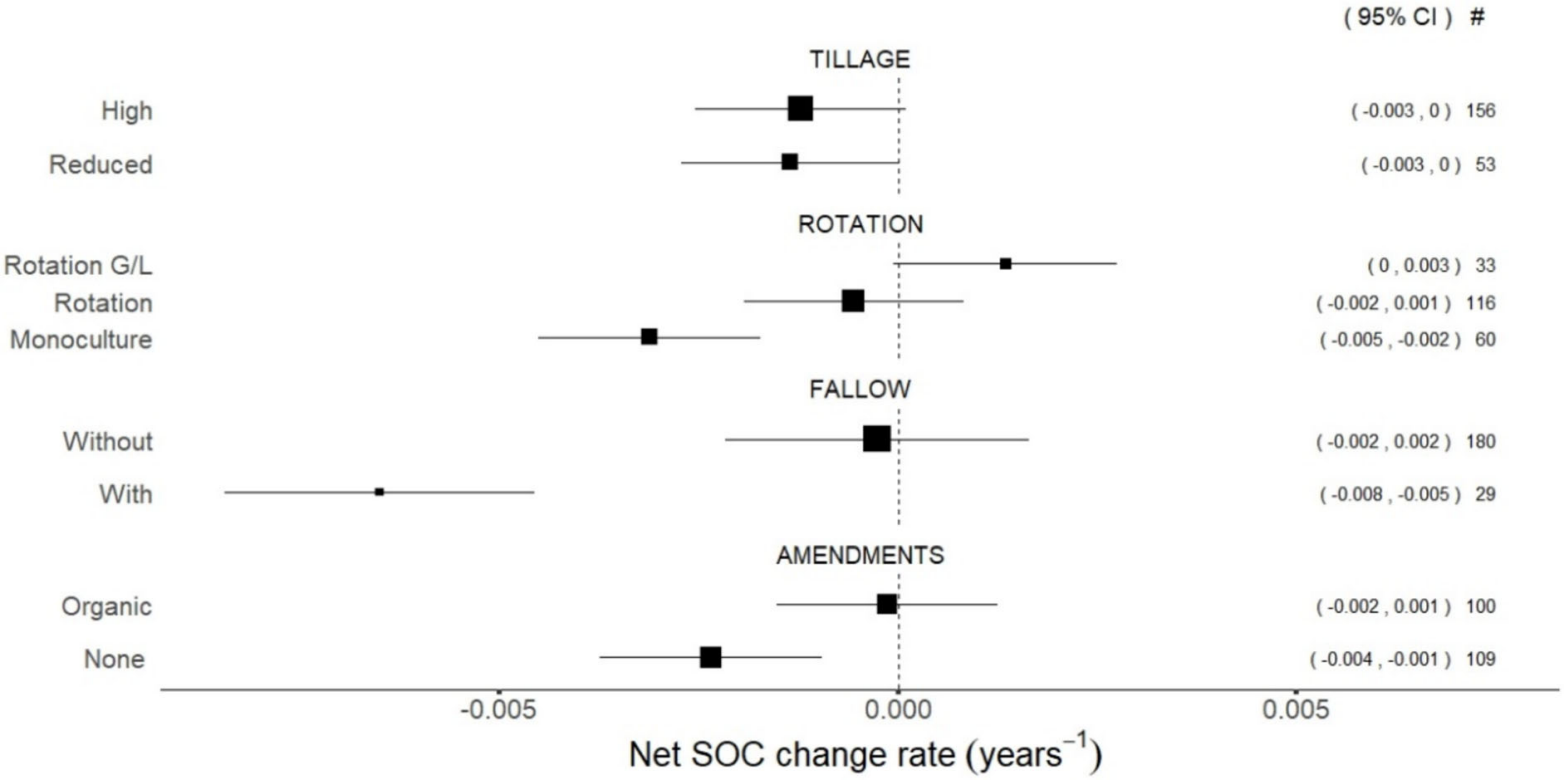
Net SOC change rate estimates for individual interventions. The size of the squares is proportional to the number of time series represented in each category, which is reported under #. Bars correspond to the 95% confidence interval of the effect, which is reported in (·)

Several of the overall effects associated with individual interventions were significantly different from zero, thus indicating that management influences SOC content in arable land (Fig. 4). However, most interventions within each group of management did not show net SOC change rates that were significantly different from each other. As an exception to this, fields practicing bare fallow (continuous or summer fallow) showed significantly lower rates of net SOC change than fields without any bare fallow periods, and fields including grasses or legumes in their rotations showed significantly higher net rates than monocultures. Overall, the highest decline rates within each management group were found for monocultures (− 0.005 to − 0.002), bare fallow (− 0.008 to − 0.005), and no use of organic amendments (− 0.004 to − 0.001). In the case of tillage, net rates of change for high and reduced alternatives were virtually the same when disregarding the effects of all other management interventions.

### Effects of paired interventions

**Fig. 5:**
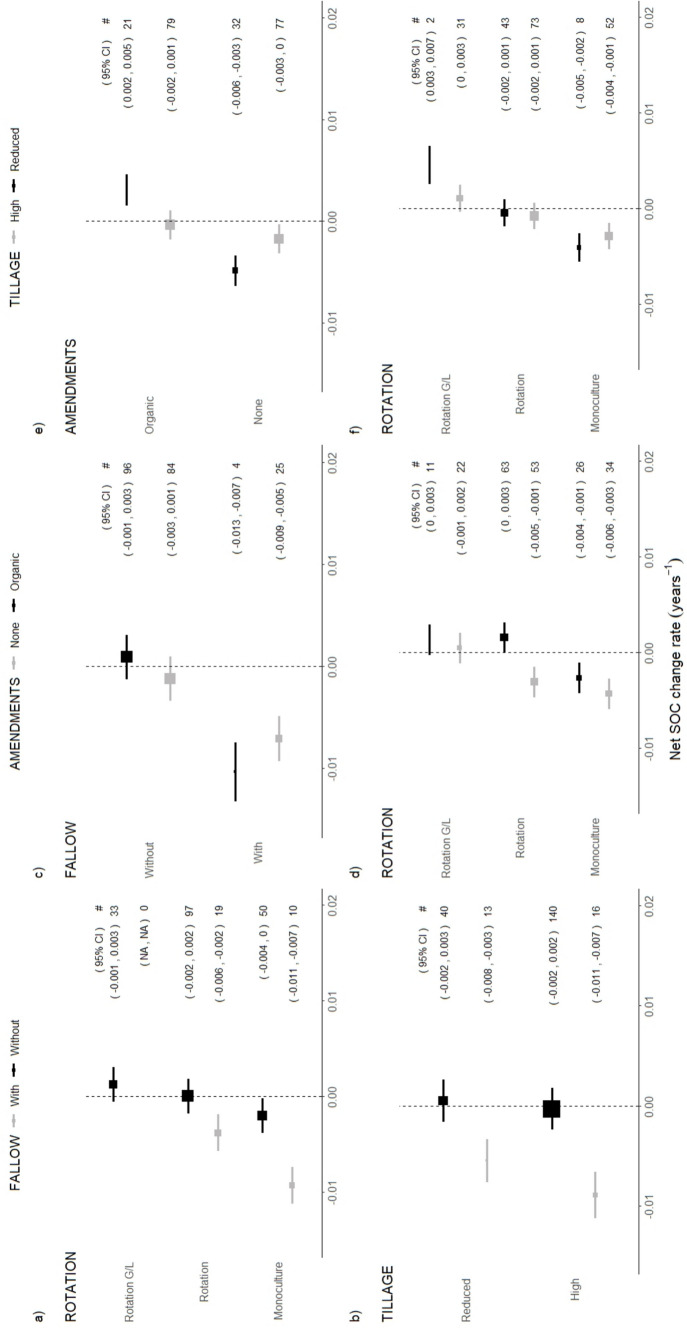
Net SOC change rate estimates across pairs of interventions. The size of the squares is proportional to the number of time series represented in each category, which is reported under #. Bars correspond to the 95% confidence interval of the effect, which is reported in (·)

Pairing interventions to strengthen effect size predictors allowed us to observe statistically significant differences across (at least some) practices within all management groups considered in the meta-analysis (Fig. 5). While most estimates of net SOC change rates across paired interventions were not significantly above zero, some estimates were significantly higher than others, thus indicating that some management suites reduced SOC loss comparatively to others. In addition, a few selected pairs of interventions showed positive SOC change rates, indicating net SOC growth over time. However, our analysis on the effect of inorganic fertiliser application (regardless of the amount) failed to capture any significant results and was relegated to the supplementary material (Fig. S5.2).

#### Tillage

Reduced tillage in combination with organic amendments or avoiding bare fallow periods had a positive effect in preventing SOC loss (Fig. 5e, b). Furthermore, reduced tillage combined with use of organic amendments showed positive net SOC change rates (0.002–0.005), thus indicating SOC growth over time. In contrast, no interaction effects were found between different tillage and crop rotation practices (Fig. 5f), except in rotations with grasses or legumes and reduced tillage, although in this case the pool of evidence consisted of only two long-term time series.

#### Rotation

Diversifying crop rotations while avoiding bare fallow showed a positive effect on net SOC change rates, which was highest for crop rotations with grasses or legumes and without bare fallow (Fig. 5a). Crop monocultures also exhibited higher rates than fields in continuous bare fallow (defined in Fig. 5a as monocultures with fallow).

Organic amendments had a significant effect in fields managed in crop rotations without grasses or legumes resulting in net positive SOC change rates (Fig. 5d). In contrast, it did not show a significant effect in crop rotations with grasses or legumes, or monocultures. Both types of rotations with organic amendments showed higher rate estimates than monocultures.

#### Fallow

The management group concerning bare fallow periods already exhibited the largest differences on SOC as a single intervention (Fig. 4). Avoiding bare fallow had significant positive effects on SOC in pairs of interventions with all other groups of management, although none resulted in net SOC change rates significantly above zero (Fig. 5a–c). While the use of organic amendments showed a decrease in the net SOC change rate estimate in fields with bare fallow, the pool of evidence consists only of four time series, which precludes any conclusions from this result (Fig. 5c).

#### Amendments

The use of organic amendments showed significant positive effects on SOC in combination with reduced tillage and rotations without grasses or legumes (Fig. 5d, e). Fields managed with organic amendments and avoiding bare fallow showed an estimated effect significantly higher than fields with bare fallow and no use of amendments. In fields without use of organic amendments, the range of the rate estimate is lower for reduced tillage than for high tillage, although the difference is not statistically significant.

### Effects of multiple interventions for SOC restoration

The data set allowed for a comparison of multiple interventions independently of the type of management. We considered five groups of management, where no interventions for SOC preservation was defined as a monoculture field experiment with high tillage, no application of organic amendments, and practicing bare continuous or summer fallow. Any other treatment category within each management group was considered to increase the number of interventions by one level. This classification of interventions for SOC preservation is consistent with the effects of the different practices on SOC change rates observed in Fig. 5.

**Fig. 6:**
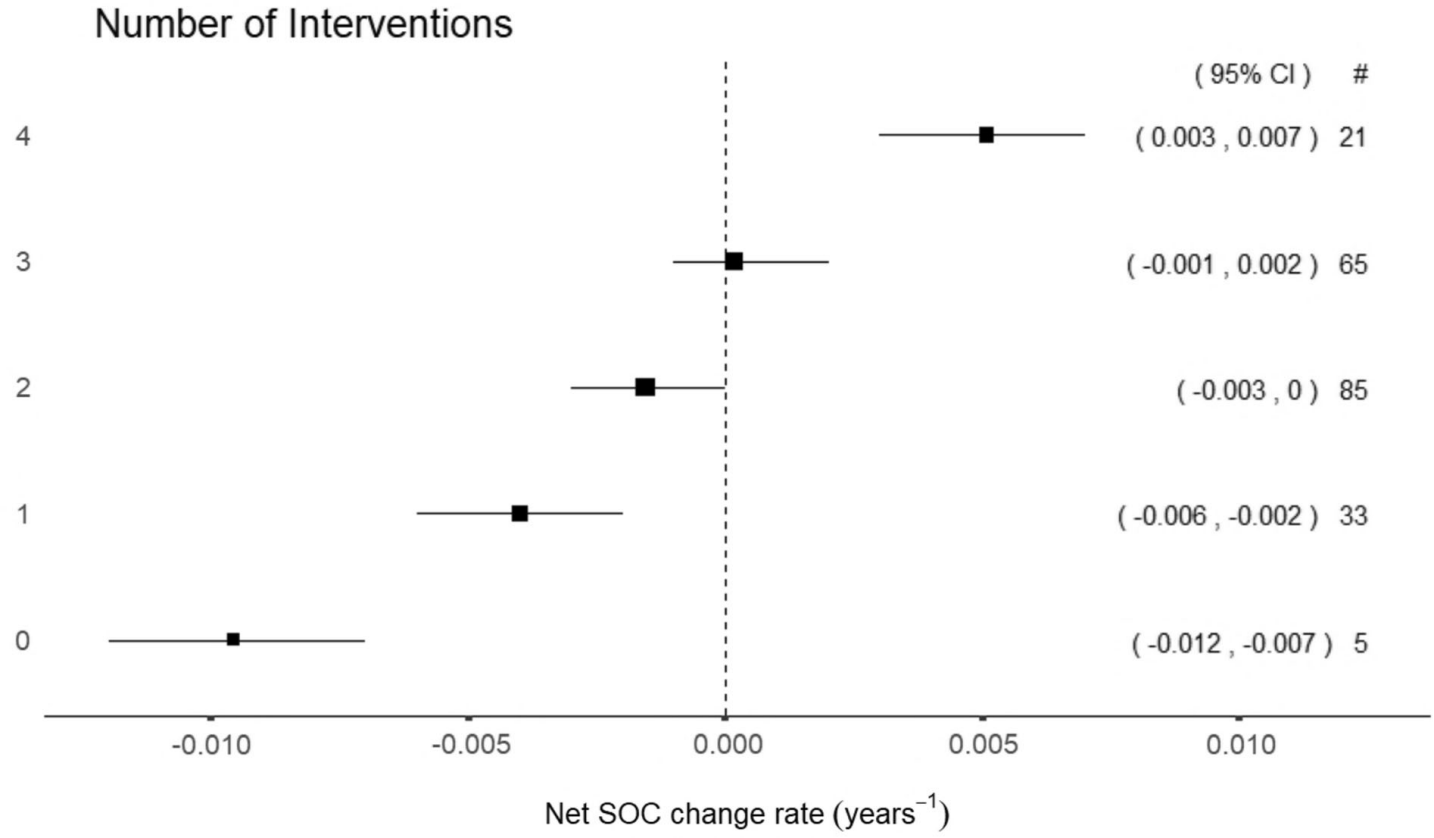
Net SOC change rate estimates for an increasing number of interventions. The size of the squares is proportional to the number of time series represented in each category, which is reported under #. Bars correspond to the 95% confidence interval of the effect, which is reported in (·)

The effect on SOC of an increasing number of interventions had a positive trend, with estimates for three and four interventions being significantly higher than those with, respectively, none, and one intervention (Fig. 6). Notably, net positive SOC change rates contributing to enhance SOC content were found in time series including interventions in all four groups of management. In fact, the sharp increase in the effect estimate for this category suggests synergistic effects of combining multiple interventions. Overall, our study supports the assertion that applying multiple, diverse interventions contributes positively to SOC restoration in agriculture. The underlying distribution of SOC change rates for each type of management intervention within an increasing number of interventions is included in the Supplementary Information file S5 (Fig. S5.7).

All files used in the meta-analysis, including source code in R, can be found in López i Losada et al. (2025). Detailed statistical output concerning all variations of the mixed effect model are provided in Supplementary Information file S6.

## Discussion

### SOC change due to management in agriculture

Fitted net rates of SOC change were negative for most long-term agricultural experiments included in our review. While this indicated that the experiment fields generally lose SOC over time, legacy effects of past land use (often not reported) could strongly affect SOC development (Guillaume et al. 2021). However, a Spearman correlation test (Table S5.4) showed no correlation between the effect size used in our meta-analysis (i.e., net SOC change rates) and initial concentrations or stocks of SOC in long-term experiments. This indicates that our approach to determine the influence of management interventions from long-term experiments on SOC through net rates of SOC change was robust to legacy effects from past land use. Therefore, our study showed that most management suites included in our meta-analysis affect SOC in arable land negatively and contribute to the declining SOC trends observed in current agriculture.

The highest declines were observed in fields practicing bare continuous or summer fallow. While not as extended in other regions, bare fallows are a common practice under semi-arid conditions particularly during the summer months, and their damaging effects on SOC is well documented (Bremer et al. 1995). Further, diversified crop rotations with grasses and legumes showed significant potential to lower SOC loss in alignment with previous studies (Englund et al. 2023). However, we found no significant difference when analysing the effects of reduced tillage and the use of organic amendments as individual interventions. Arguably, disregarding interactions with other management groups made effect estimates for individual interventions relatively weak, given that net SOC change rates under any reported treatment also included a broad range of practices under the other management groups.

Our meta-analysis approach on multiple interventions allowed us to estimate combined effects of simultaneous practices. When considering paired interventions across management groups, reduced tillage (Thapa et al. 2023), crop rotations (Englund et al. 2023), use of amendments (Bai et al. 2018), and avoiding bare fallow, all showed a significant potential to lower SOC loss in alignment with previous studies (Han et al. 2016; Bolinder et al. 2020; Lessmann et al. 2022). Increasing rates of inorganic fertiliser application has also been shown to enhance SOC (Lu et al. 2011; Han et al. 2016), though our meta-analysis approach of combined interventions with only two categorical values (with/without) assigned to different inorganic fertilisation regimes was insufficient to capture this effect (Fig. S5.2). A substantially larger pool of evidence would have been required to categorise experiments across a gradient of application levels of inorganic fertiliser.

In addition, our results showed significant asymmetries in the SOC responses of several interventions when evaluated in combination with other management groups. For instance: (a) reduced tillage showed positive effects in combination with organic amendments but not without them, (b) only rotations without grasses or legumes benefitted from the addition of organic amendments, and (c) the net SOC change rate estimate for the combination of interventions from all groups suggests synergistic effects. These findings indicate that the extent to which individual interventions affected SOC change rates was influenced by other management decisions. As treatments across groups are not mutually exclusive (e.g., reducing tillage and applying organic amendments) and in fact are often applied in combination (Francaviglia et al. 2023), this stresses the relevance of evaluating management comprehensively. Indeed, SOC trends from studies on single management groups or several of them separately cannot be generalised without introducing assumptions on the additionality of the effects, implying that simultaneous interventions from different management groups caused independent effects. This assumption has been broadly identified as a limitation to estimating SOC development on agricultural land (Goglio et al. 2015; Bolinder et al. 2020; Lessmann et al. 2022). Overall, our analysis shows that individual interventions would only contribute to reducing SOC loss, while combining good practices across all four groups of management could effectively restore SOC content at a yearly rate of 0.0059 on average.

While our data included both positive (76 datasets) and negative change rates (138 datasets), Fig. 2 indicates that positive rates were overrepresented among those with poor fit. A Chi-squared test of positive and negative rates in association with high and low R-square (above and below 0.3) confirmed that the difference was significant. Gain and loss mechanics of SOC are not symmetric (Sanderman and Baldock 2010), and the possibility of SOC eventually plateauing after growth is not considered within our log-linear model, which may explain the better fit of our model to declining trends. In contrast, validation of the underlying data suggests that dynamic models may perform better for increasing SOC trends (Coleman et al. 1997). However, our approach should not be confounded with physical soil carbon modelling, as it instead provides statistically significant effect sizes based on an experimental pool of evidence.

### SOC sequestration from sustainable soil management

Claims of SOC sequestration in the literature most often do not discriminate between C gains relative to a control and absolute increases in SOC (Don et al. 2024). While relative gains can prevent further SOC loss, only absolute gains result in negative emissions and enhancement of soil ecosystem services from the present-day baseline. Our inclusion criteria address three main drawbacks identified from the experimental evidence supporting previous work. First, studies rarely discriminate in their pool of evidence against experiments that are too brief (Vicente-Vicente et al. 2016), which is detrimental for observing substantial SOC differences occurring over decades. Second, net and relative SOC change is often confounded by combining data from long-term time series and experiments that measure differences relative to a control at a single sampling date (Bai et al. 2018; Gocke et al. 2023; Joshi et al. 2023). Third, studies often focus on management practices in isolation (Lessmann et al. 2022), which precludes estimation of combined effects. These issues are ultimately detrimental for predicting reliable estimates of SOC changes from agricultural management (Sanderman and Baldock 2010; Haddaway et al. 2016), and may have contributed to contrasting outcomes (Vandenbygaart et al. 2008) and apparent inconsistencies across previous reviews (Bolinder et al. 2020).

By overcoming these challenges, our statistical approach based on long-term experiments allowed us to evaluate whether SOC increases or decreases in the topsoil layer of agricultural land due to a range of management options considered by farmers. In addition, our pool of evidence comprises a wide array of experimental sites and our meta-analysis approach including random effects allows us to extract conclusions on the influence of agricultural management beyond site-specific biases. In spite of a restricted pool of available data to comply with our inclusion criteria, and high levels of spatiotemporal variability in SOC that are often attributed to exogenous factors such as climatic variables or elevation (Wang et al. 2021), our study provided sufficient evidence on how management interventions influence SOC development over the long-term. Our meta-analysis of management practices showed substantial power with an evidence base spanning a wide range of climate conditions, soil textures, initial SOC content and experiment durations. Incidentally, the duration of management (which varied widely), and the use of land prior to establishment (which was not recorded), are both aspects governing non-trivial steady-state assumptions affecting the robustness of SOC dynamic modelling approaches (Petersen et al. 2013; Joensuu et al. 2021).

Data availability was still a decisive factor in our meta-analysis, as it determined the level of aggregation of our intervention categories. We deemed the amount of data gathered in this review insufficient, for instance, to study net SOC change rates separately in continuous vs. summer bare fallows, or under different levels of fertiliser application (Nilsson et al. 2023). In addition, SOC measurements in deeper soil layers (i.e., under 30 cm) were less frequently recorded in long-term experiments, which limited our analysis to the topsoil layer despite available evidence showing the influence of agricultural management in the subsoil (Skadell et al. 2023). Our evidence base would have been further limited had we not decided to include time series with SOC recordings in both concentrations (151 time series across 17 sites) and stocks (64 time series across 8 sites).

Combining data on stocks and concentrations could in principle confound the outcomes of our analysis, particularly as estimates of SOC development are sensitive to methodological choices (Wendt and Hauser 2013). However, bulk density is either motivated or assumed constant in half of the time series in which SOC development was reported in stocks and their individual rates of change are hence indicative of change in SOC concentrations (Lugato et al. 2007; Persson et al. 2008; Buysse et al. 2013; Congreves et al. 2015). Besides, our mixed effects model considers random effects across experimental sites, which should be able to capture site-specific experimental and methodological bias. Further analysis of bulk density development over time across management systems was not possible given a general lack of records in our list of included studies.

### Implications for research, policy, and practice

Preservation and restoration of SOC in agricultural land is an important component of environmental policymaking concerned with climate change mitigation, nature restoration and sustainable agriculture (Bradford et al. 2019; Boix-Fayos and de Vente 2023). Enabling simple and reliable estimation of SOC development in agricultural land is a relevant step towards understanding the societal costs and benefits of measures promoting good agricultural practices for soil, thus contributing with scientific evidence for policymaking. Our meta-analysis of long-term experimental evidence allows reliable conclusions on practices that farmers can apply to enhance SOC content in the topsoil layer, i.e., that multiple and diverse interventions on tillage, organic amendments, and crop rotations can effectively restore depleted SOC levels in arable land in regions with favourable climatic conditions to grow wheat.

Estimating SOC accrual rates at regional or larger scales often relies on statistical analysis of a broad pool of agricultural experiments or process-based SOC modelling (Goglio et al. 2015). Complex SOC modelling approaches recommended in the higher tiers of the IPCC guidelines for national carbon inventories may require extensive soil expertise and data for calibration, and still provide rates of change that are highly sensitive to model assumptions (Joensuu et al. 2021). Our simple statistical approach addresses drawbacks associated with the evidence base from previous statistical studies and predicts considerably narrow confidence intervals on mean SOC development across arable fields within a wide climatic range. Given that rates of change used in this study are relative to initial SOC content and this varied widely across individual experiments (0.8–6.9% in time series reporting SOC concentrations), our results imply large differences in the C sequestration potential for climate change mitigation from management interventions across regions with different SOC levels. At the same time, prediction intervals from our meta-analysis showed broad, indistinct ranges (Fig. S5.5), meaning that positive effects were only observed on averages across large scales, while it remains uncertain whether an individual farmer would benefit from management interventions due to the natural variability of SOC from exogenous factors.

While enhancing SOC in the topsoil layer is itself a valuable goal for society to secure the ecosystem services that support agriculture, deeper soil layers concentrate substantial SOC and their response to agricultural management may differ from the topsoil (Dal Ferro et al. 2020; Hicks Pries et al. 2023). Widespread SOC measurements in the deep soil in field experiments could in the future give a fuller insight of the C sequestration potential of management interventions. In addition, a life cycle perspective coupled to our results could build understanding of the relative importance for climate change mitigation from other aspects not included in this study such as changes in operations, input requirements, yields, and N_2_O emissions (Brady et al. 2015; Oldfield et al. 2019; Jordon et al. 2022).

Future updates of this review could improve the level of resolution of the current analysis by enlarging its pool of evidence, particularly in relation to the combination of multiple practices promoting SOC (Jordon et al. 2022; Vendig et al. 2023). To this end, a recent initiative to map SOC across long-term field experiments in North America should provide additional data points to time series from a substantial number of experiments, thereby aligning with our inclusion criteria (Peng et al. 2023). These additional experiments incorporate a wide range of combinations of practices with positive effects on SOC, such as reduced tillage, diverse crop rotations, and cover crops, which would allow for finer and more comprehensive estimates of SOC change rates from management suites promoting SOC preservation in agricultural land.

## Supplementary Information

Below is the link to the electronic supplementary material.

Supplementary file1 (XLSX 92 KB)

Supplementary file2 (XLSX 466 KB)

Supplementary file3 (XLSX 35 KB)

Supplementary file4 (DOCX 20 KB)

Supplementary file4 (DOCX 671 KB)

Supplementary file4 (XLSX 16 KB)

## Data Availability

A list of included studies is provided in Supplementary Information file S1. A map of all studies resulting from the review search is provided in file S2. File S3 contains a summary of regression statistics for all time data series with a log-linear curve fit. R scripts for all analyses together with auxiliary data files are publicly available (López i Losada et al. 2025). Search strings are provided in file S4. Appendix S5 includes supplementary Figures and Tables. Summary statistics for all variations of the mixed effect model, irrespective of significance, are provided in file S6.
