# Supplementary material for: Synergistic effects of multiple “good agricultural practices” for promoting organic carbon in soils: A systematic review of long-term experiments
Source: Ambio. 2025 May 27;54(11):1715–28. doi: 10.1007/s13280-025-02188-8 (PMC12480305; doi:10.1007/s13280-025-02188-8)
Supplement: Supplementary file 4 — Supplementary file4 (DOCX 20 KB) [file 13280_2025_2188_MOESM4_ESM.docx]

**Supplementary Information file 5**

**Search strings**

In this review we used results extracted from previously published systematic reviews, that originated in a systematic map database on farming management and SOC (Haddaway *et al.*, 2015) and added data from more recent reviews on tillage (Haddaway *et al.*, 2017) and crop rotation (Land *et al.*, 2017; Formas, 2021). Thus, the resulting searches include publications until 2019.

1. *Original systematic map search (Haddaway et al., 2015)*

Searches of 15 academic databases were undertaken as part of the published systematic map in September 2013. These academic database searches were supplemented by searches for grey literature via web search engines and organisational websites, and by searches of the bibliographies of 127 relevant reviews and meta-analyses identified during the course of the systematic map. A search update was undertaken for publications during 2013-2015. Only English language search terms will be used but all articles identified in Danish, English, French, German, Italian, Russian, and Swedish were included.

**Search string:**

soil* AND (arable OR agricult* OR farm* OR crop* OR cultivat*) AND (till* OR “direct drill*” OR fertili* OR “bio*solid*” OR organic OR manur* OR sewage OR compost* OR amendment* OR biochar* OR digestate* OR “crop residue*” OR “crop straw*” OR mulch* OR “crop rotat*” OR break crop* OR grass OR clover ley* OR legume* OR “bioenergy crop*” OR “cover crop*” OR “grass clover” OR “crop* system*” OR “winter crop*” OR “spring crop*” OR “summer fallow*” OR “catch*crop*” OR intercrop* OR conservation) AND (“soil organic carbon” OR “soil carbon” OR “soil C” OR “soil organic C” OR SOC OR “carbon pool” OR “carbon stock” OR “carbon storage” OR “soil organic matter” OR SOM OR “carbon sequestrat*” OR “C sequestrat*”)

In Google Scholar the following search string was used and the first 1,000 records downloaded for both title and full text searches using a method described in Haddaway et al. [19]:

*soil AND carbon AND (till* OR “direct drill*” OR fertili* OR manur OR compost* OR amendment* OR biochar* OR digestate* OR “crop residue*” OR “crop straw*” OR mulch* OR “crop rotat*” OR grass OR legume* OR “bioenergy crop*” OR “cover cropOR “crop* system*” OR ~~“summer~~ fallow*~~”~~ OR “catch*crop*” OR intercrop* OR conservation)*

1. *Tillage review update (Haddaway et al., 2017)*

A search update was undertaken in September 2015 to capture research published since the original search in September 2013. The update was restricted to four academic databases, Academic Search Premier, Pub Med, Scopus, Web of Science (Web of Science Core Collection, BIOSIS Citation Index, Chinese Science Citation Database, Data Citation Index, SciELO Citation Index), and one academic search engine, Google Scholar. Only English language search terms was used for the update, but any articles identified in Danish, English, French, German, Italian, and Swedish were included. In both update reviews, the number of citation databases included was reduced given that large numbers of duplicates were identified during the searches performed for the systematic map.

**Search string**

The following search string was used in the academic databases mentioned above and was adapted from the original string used in the published systematic map [36] to identify specifically tillage research and restricted to the period since the original search was undertaken (September 2013):

soil* AND (arable OR agricult* OR farm* OR crop* OR cultivat*) AND (till* OR “no till*” OR “reduced till*” OR “direct drill*” OR “conservation till*” OR “minimum till*”) AND (“soil organic carbon” OR “soil carbon” OR “soil C” OR “soil organic C” OR SOC OR “carbon pool” OR “carbon stock” OR “carbon storage” OR “soil organic matter” OR SOM OR “carbon sequestrat*” OR “C sequestrat*”)

In Google Scholar the following search string was used:

soil AND carbon AND (till OR tillage OR “reduced tillage” OR “conservation tillage” OR “no tillage” OR “direct drill” OR “minimum till*”)

1. *Crop rotation review update (Land et al., 2017; Formas, 2021):*

An update in August 2019, was restricted to four bibliographic databases: 1) Academic Search Premier (https://www.ebscohost.com/academic/academic-search-premier), 2) Pub Med (https://www.ncbi.nlm.nih.gov/pubmed), 3) Scopus (https://www.elsevier.com/solutions/scopus), 4) Web of Science Core Collection (<http://apps.webofknowledge.com/>), and one academic search engine, Google Scholar (https://scholar.google.se)

**Search string:**

*soil* AND (arable OR agricult* OR farm* OR crop* OR cultivat*) AND (legume$ OR pulse$ OR “green-manure” OR alfalfa$ OR lupin$ OR bean$ OR pea$ OR lentil$ OR clover OR soy OR soybean$ OR perennial$ OR grass* OR ley$ OR permaculture OR rotation OR monoculture OR “mono culture”) AND (“soil organic carbon” OR “soil carbon” OR “soil C” OR “soil organic C” OR SOC OR “carbon pool” OR “carbon stock” OR “carbon storage” OR “soil organic matter” OR SOM OR “carbon sequestrat*” OR “C sequestrat*”)*

[the underlined text indicates modifications to the original systematic map search string]

**References**

Formas, 2021. Växtföljders påverkan på inlagring av organiskt kol i jordbruksmark En systematisk översikt och samhällsekonomisk analys. <https://formas.se/analys-och-resultat/rapporter/2021-04-01-vaxtfoljders-paverkan-pa-inlagring-av-organiskt-kol-i-jordbruksmark.html>.

Haddaway, N.R., Hedlund, K., Jackson, L.E., Kätterer, T., Lugato, E., Thomsen, I.K., Jørgensen, H.B., Isberg, P.-E., 2017. How does tillage intensity affect soil organic carbon? A systematic review. Environmental Evidence 6, 30.

Haddaway, N.R., Hedlund, K., Jackson, L.E., Kätterer, T., Lugato, E., Thomsen, I.K., Jørgensen, H.B., Söderström, B., 2015. What are the effects of agricultural management on soil organic carbon in boreo-temperate systems? Environmental Evidence 4, 23.

Land, M., Haddaway, N.R., Hedlund, K., Jørgensen, H.B., Kätterer, T., Isberg, P.-E., 2017. How do selected crop rotations affect soil organic carbon in boreo-temperate systems? A systematic review protocol. Environmental Evidence 6, 9.
