# Supplementary material for: Synergistic effects of multiple “good agricultural practices” for promoting organic carbon in soils: A systematic review of long-term experiments
Source: Ambio. 2025 May 27;54(11):1715–28. doi: 10.1007/s13280-025-02188-8 (PMC12480305; doi:10.1007/s13280-025-02188-8)
Supplement: Supplementary file 5 — Supplementary file4 (DOCX 671 KB) [file 13280_2025_2188_MOESM5_ESM.docx]

**Supplementary Information**

File S1 Database of included publications.xlsx

File S2 Aggregated metadatabase.xlsx

File S3 Summary of statistics - regression.xlsx

File S4 search strings.docx

File S5 Appendix of tables and figures

File S6 Summary of statistics - meta-analysis.xlsx

**Ambio**

Supplementary information file S5

*This supplementary information has not been peer reviewed*

Title: **Synergistic effects of multiple “good agricultural practices” for promoting organic carbon in soils: A systematic review of long-term experiments.**


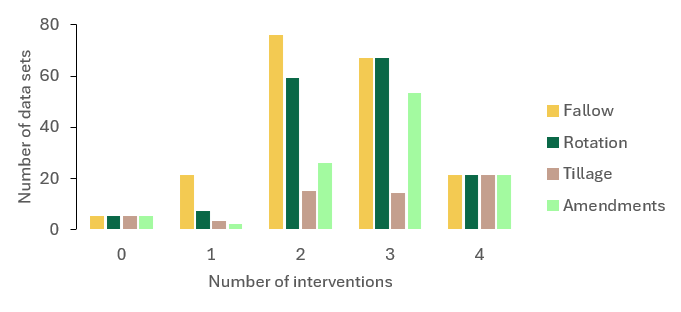


*Figure S5.1 – Number and type of intervention across the long-term experimental data sets
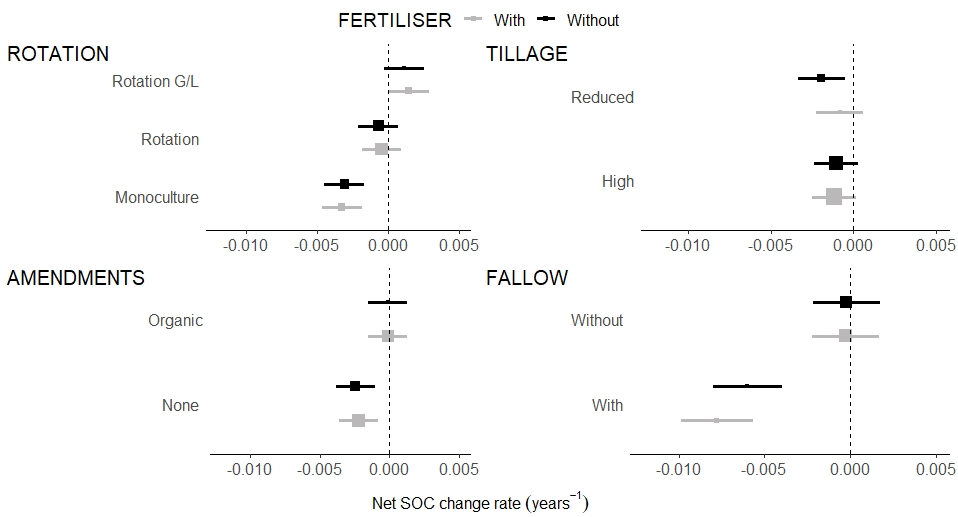
*

*Figure S5.2 – Net SOC change rate estimates across pairs of interventions including inorganic fertiliser application. Square sizes represent the number of time series within a pair, whereas bars indicate the 95% interval of confidence of the estimate.*


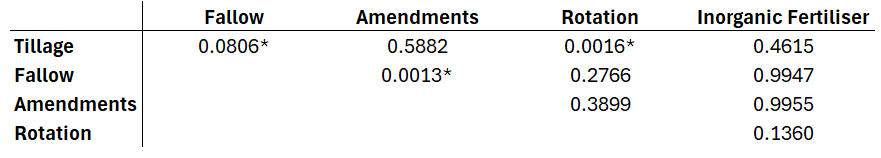


*Table S5.3 – P-values for Chi-squared tests on paired management groups. * indicates p-value under 0.1*


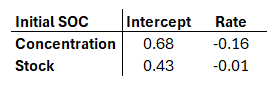


*Table S5.4 Spearman correlation coefficients between initial SOC content and, respectively, fitted intercept and rate coefficients for the log-linear model of individual time series. Test performed separately for experiments with measurements in concentrations and stocks.*

*
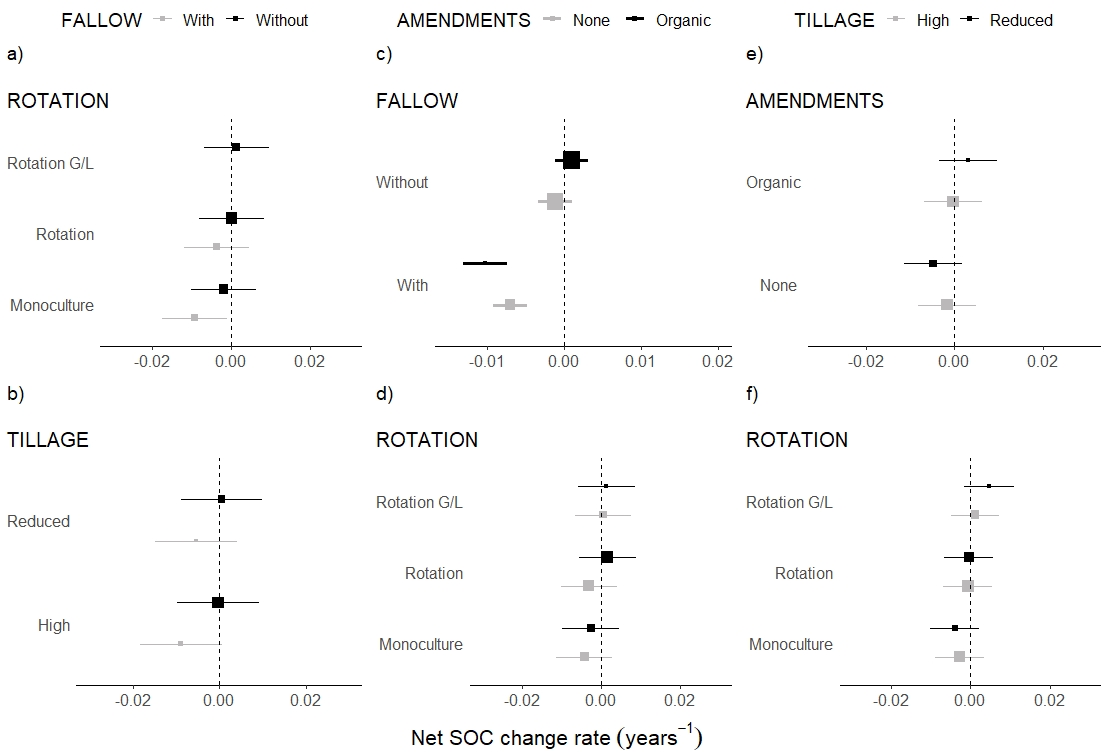
*

*Figure S5.5 – Net SOC change rate estimates and intervals of prediction across pairs of interventions. Square sizes represent the number of time series in each category, whereas bars indicate the 95% interval of prediction of the estimate.*

*
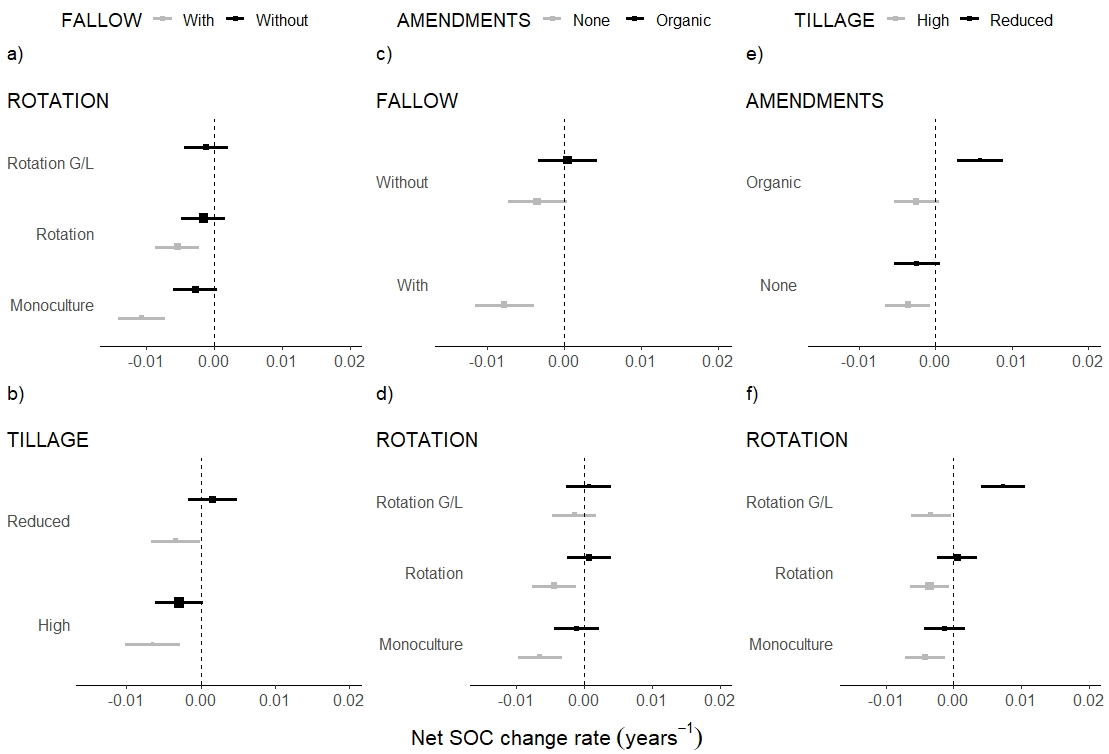
*

*Figure S5.6 – Sensitivity analysis of net SOC change rate estimates across pairs of interventions only considering studies with high validity. Square sizes represent the number of time series within a pair, whereas bars indicate the 95% interval of confidence of the estimate.*

*
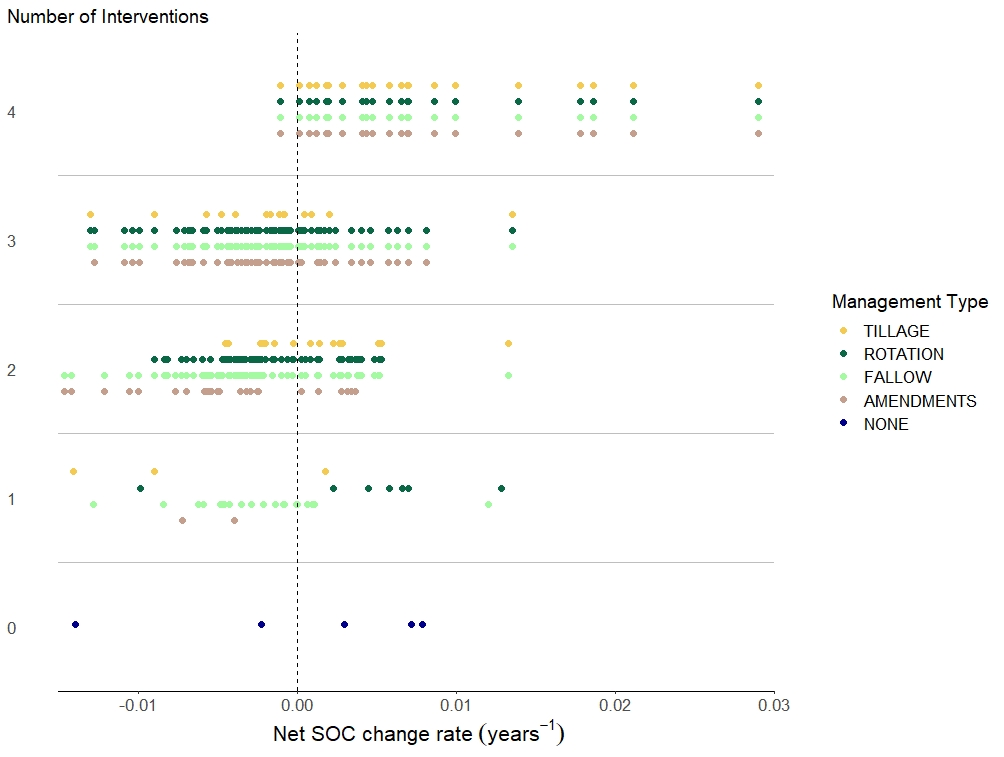
*

*Figure S5.7 – Distribution of net SOC change rates in long-term experiments for each type of management intervention within an increasing number of interventions*

*
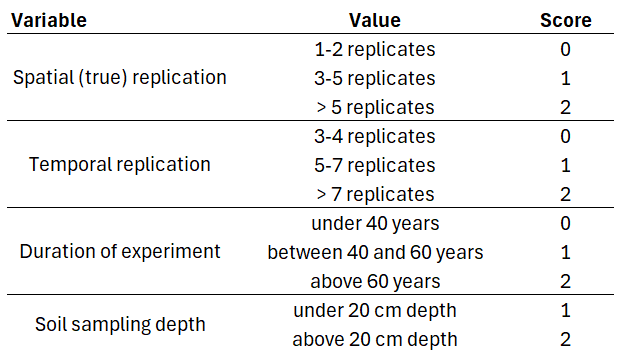
*

*Table S5.8 – Criteria for critical appraisal of long-term time series*
